# Ludwig's Angina in a Pediatric Patient: A Case Report

**DOI:** 10.7759/cureus.81131

**Published:** 2025-03-25

**Authors:** Ana Sofia Silva, Maria Pereira Fernandes, Nina Berdianu, Helena Sofia Sousa, Florbela Cunha

**Affiliations:** 1 Pediatrics, Unidade Local de Saúde de Santa Maria - Hospital de Santa Maria, Lisbon, PRT; 2 Pediatrics, Unidade Local de Saúde Estuário do Tejo - Hospital de Vila Franca de Xira, Vila Franca de Xira, PRT; 3 Pediatrics, Unidade Local de Saúde de São José - Hospital de Dona Estefânia, Lisbon, PRT; 4 Paediatrics, Unidade Local de Saúde Estuário do Tejo - Hospital de Vila Franca de Xira, Vila Franca de Xira, PRT

**Keywords:** antibiotic therapy, ludwig’s angina, odontogenic infection, submandibular gland, surgical drainage

## Abstract

Ludwig's angina (LA) is a life-threatening, rapidly progressive infection of the submandibular, sublingual, and submental spaces, often caused by odontogenic infections. We report the case of an eight-year-old previously healthy girl who developed LA following untreated dental caries. She presented with a four-day history of odontalgia, left hemifacial edema, and trismus. Despite an initial course of oral amoxicillin-clavulanate, her condition worsened, leading to hospital admission. Examination revealed fever (38.1°C), painful left submandibular swelling, trismus, and molar caries. Laboratory tests showed leukocytosis, neutrophilia, and elevated C-reactive protein (CRP), suggesting severe infection. Intravenous amoxicillin-clavulanate and clindamycin were initiated, but symptoms progressed with increasing pain, sublingual swelling, and worsening trismus. A cervical CT confirmed an abscess in the left sublingual space with extensive periradicular lytic lesions. Urgent surgical intervention included extraction of teeth 36 and 75 and abscess drainage, which revealed purulent material. Postoperatively, the patient improved with a 14-day antibiotic course, resolving fever and swelling. This case underscores the importance of early recognition and prompt treatment of LA in pediatric patients. Multidisciplinary management is essential for optimal outcomes. Additionally, vigilant dental care and good oral hygiene are key to preventing odontogenic infections and severe complications.

## Introduction

Ludwig's angina (LA) is a life-threatening, rapidly progressive soft tissue infection involving the floor of the mouth and surrounding structures, often resulting from odontogenic infections that spread to deeper neck spaces. It is typically triggered by untreated or poorly managed dental infections, particularly dental caries [1].

LA is a cellulitis of the submandibular, sublingual, and submental spaces, often occurring bilaterally. The infection is commonly caused by pathogens from the oral cavity, including *Streptococcus *species (particularly* Streptococcus pneumoniae* and *Streptococcus viridans*), *Staphylococcus aureus* (including methicillin-resistant *S. aureus*), and anaerobic bacteria such as *Bacteroides *and *Fusobacterium *species. These bacteria, often in polymicrobial combinations, can cause rapid progression of the condition. Early diagnosis and prompt intervention are critical to prevent severe complications, such as airway obstruction, sepsis, and further deterioration [1,2].

This report presents the case of a previously healthy eight-year-old girl who developed LA following untreated dental caries. Despite initial antibiotic treatment, her condition worsened, leading to urgent surgical intervention.

## Case presentation

An eight-year-old girl with no relevant past history presented to the emergency department with a four-day history of tooth pain, swelling on the left side of her face, and difficulty opening her mouth. She was treated with oral amoxicillin-clavulanate (AMC) for two days upon the recommendation of her dentist, but she was unable to take the medication orally due to pain.

On physical examination, the patient was febrile (38.1°C) and exhibited significant painful swelling with erythema and warmth in the left submandibular region. There was also trismus and extensive molar caries. Laboratory results revealed leukocytosis, neutrophilia, and elevated C-reactive protein (CRP) (Table 1). Blood culture was negative.

**Table 1:** Laboratory parameters of the patient and reference ranges Elevated leukocytes, neutrophils, and CRP levels indicate an ongoing inflammatory or infectious process.

Parameters	Patient Values	Reference range
Hemoglobin	13.3 g/dL	11.5-15 g/dL
Leukocytes	17,700/µL	4,500-13,500/µL
Neutrophils	12,600/µL	1,500-8,000/µL
Lymphocytes	3,200/µL	1,500-6,800/µL
Platelets	307,000/µL	150,000-450,000/µL
C-reactive protein (CRP)	4.86 mg/dL	<1 mg/dL

**Figure 1:**
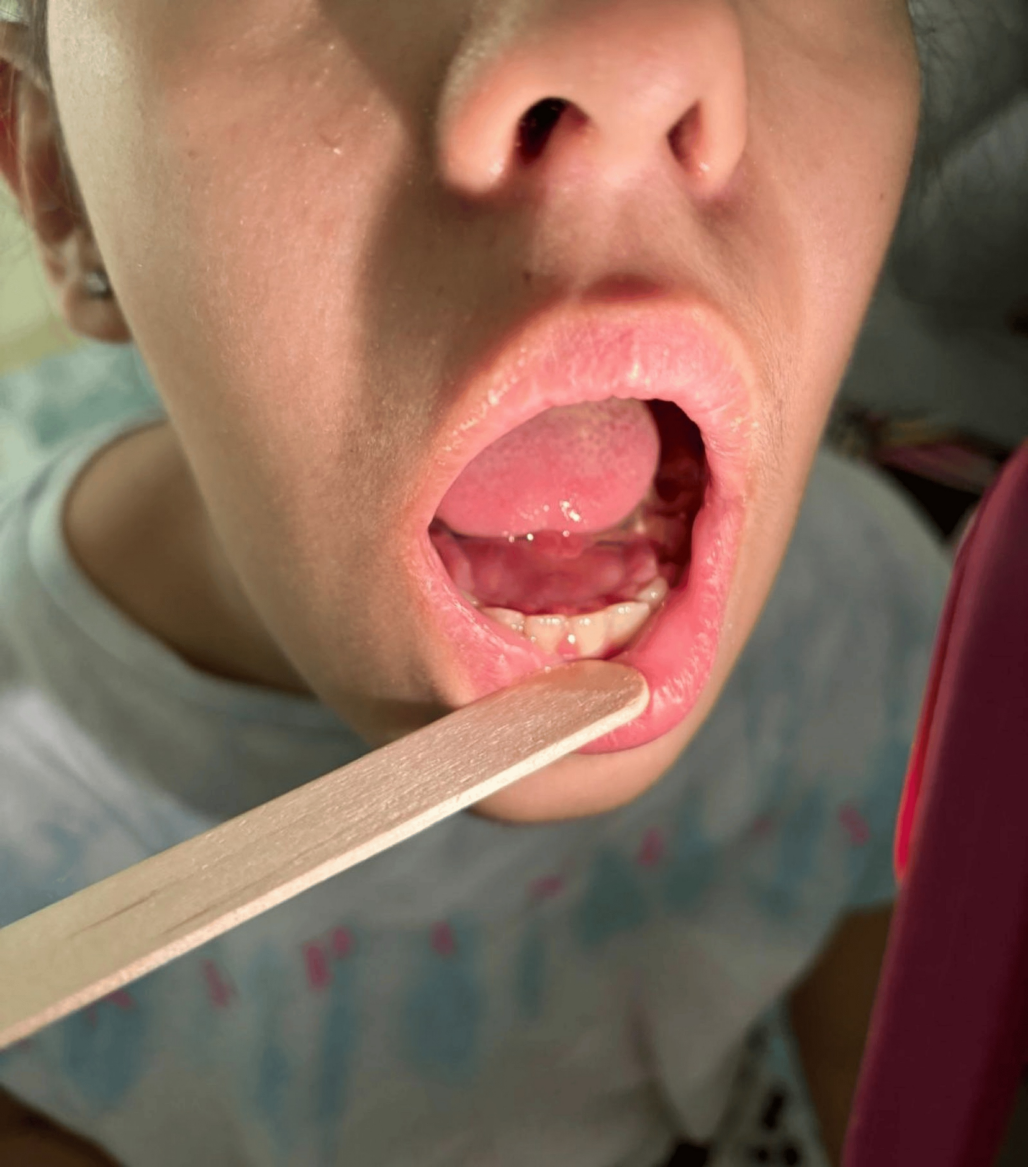
Prominent edema of the sublingual floor, consistent with the signs of a deep space infection in the context of Ludwig's angina

Given the severity of the clinical presentation, the patient was admitted to the hospital and started on intravenous AMC and clindamycin. Despite the initial treatment, the patient’s condition worsened. She developed increased pain, swelling of the sublingual floor, worsening trismus, sialorrhea, and an increase in local inflammatory signs, suggesting the progression of the infection to deeper neck spaces (Figure 1).

**Figure 2:**
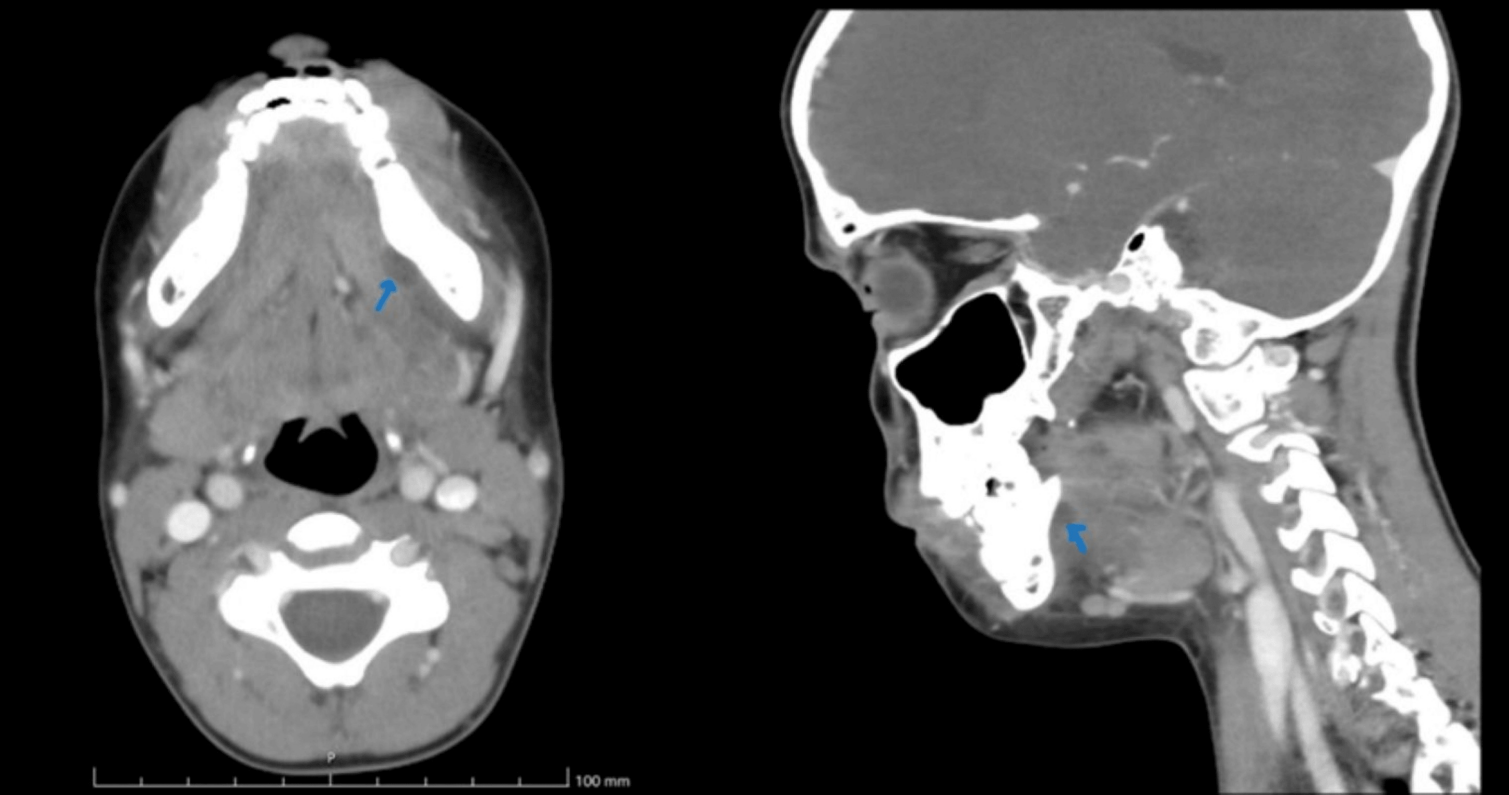
CT scan (Day 3) showing enlargement of the left submandibular gland with hypodensity in the left sublingual space near tooth 36 (blue arrows). Extensive crown erosion and periradicular lytic lesions are also observed, consistent with an odontogenic abscess and Ludwig's angina.

A cervical CT scan showed enlargement of the left submandibular gland with hypodensity in the left sublingual space near tooth 36. Additionally, there was also extensive destruction of the crown of tooth 36 and periradicular lytic lesions, consistent with an abscess formation secondary to odontogenic infection (Figure 2). These findings were highly suggestive of LA, characterized by the spread of infection to the submandibular and sublingual spaces.

Urgent consultation with the stomatology department led to the decision to extract the affected teeth (36 and 75) under general anesthesia. The procedure revealed purulent material, confirming the diagnosis of an odontogenic abscess with associated submandibular involvement. The extraction of the teeth and drainage of the abscess resulted in an improvement in the patient’s symptoms. 

Following the surgical intervention, the patient continued a 14-day regimen of dual antibiotic therapy (AMC and clindamycin) along with topical antiseptic treatment for wound care. Over the following days, her fever subsided, and the swelling gradually diminished. CRP decreased to 2.54 mg/dL, and leukocyte count dropped to 10.000/µL, indicating a positive response to treatment and improvement in the inflammatory status. The trismus improved, and there was no recurrence of sialorrhea.

## Discussion

LA is a rapidly progressing and potentially fatal condition, often resulting from untreated or inadequately treated dental infections. In pediatric patients, early diagnosis and intervention are critical to prevent severe complications such as airway obstruction, sepsis, and the spread of infection to deeper neck structures [1-3]. In this case, the progression of an odontogenic infection to LA was facilitated by the patient’s dental caries and inadequate initial antibiotic therapy. The clinical signs of fever, localized swelling, trismus, and pain are classic presentations of this condition, which require immediate attention [3,4].

Imaging plays a crucial role in the diagnosis and management of LA. CT scans can help determine the extent of the infection, identify affected structures, and guide treatment decisions. In this case, the CT scan confirmed the involvement of the submandibular gland and surrounding spaces, which was essential in planning the surgical intervention. Typical findings on CT scans include soft tissue swelling, deep fascial edema, and rim-enhancing abscesses. Severe cases may show subcutaneous emphysema, internal jugular vein thrombosis, or mediastinal extension [4,5].

Additionally, the presence of sublingual edema served as a critical clinical clue, reinforcing the need for imaging and intervention. Prompt surgical management, including tooth extraction and abscess drainage, is a cornerstone of treatment [4]. This case highlights the importance of aggressive debridement and drainage to relieve the pressure from the infected tissues, which significantly contributed to the patient’s positive outcome.

Although LA is a rare complication, its severity underscores the critical importance of early recognition, timely antibiotic therapy, and prompt surgical intervention to prevent life-threatening complications, as demonstrated in this case. Severe odontogenic infections should be closely monitored, and multidisciplinary management is essential for optimal outcomes [4]. Early intervention can result in a favorable recovery. However, despite prompt and appropriate treatment, the mortality rate remains approximately 8% [6]. Molar caries predispose to LA angina, highlighting the need for vigilant dental care in at-risk individuals. Maintaining proper oral hygiene is crucial for preventing dental infections.

## Conclusions

The present case highlights the critical importance of early recognition and prompt intervention in LA, particularly in pediatric patients who are more vulnerable due to their smaller airway dimensions. Delayed diagnosis and inadequate initial treatment can lead to rapid disease progression, increasing the risk of airway compromise, sepsis, and systemic complications. This case underscores the necessity of a multidisciplinary approach, where collaboration between pediatricians and dental specialists is essential to ensure timely diagnosis, imaging, and appropriate management, including antibiotic therapy and surgical intervention.

Furthermore, this report emphasizes the role of dental health in preventing severe infections such as LA. Untreated dental caries remain a major risk factor for odontogenic infections that may spread to deep neck spaces. Public health efforts should focus on reinforcing preventive dental care, promoting regular dental check-ups, and educating caregivers on the importance of early dental treatment. Strengthening awareness and implementing preventive strategies could significantly reduce the incidence of severe odontogenic infections and their potentially life-threatening complications.
